# A Call to Transform Maternal and Child Health Mentorship to Build Inclusivity, Honor Diversity of Experiences, and Tackle the Root of Health Disparities

**DOI:** 10.1007/s10995-021-03267-4

**Published:** 2021-11-13

**Authors:** Kathryn E. Mishkin, Grace Guerrero Ramirez, Anne Odusanya, Benjamin Kaufman

**Affiliations:** 1 Professional Development Committee, Maternal and Child Health Section, American Public Health Association, Washington, DC USA; 2 Workforce Development & Capacity Building, Association of Maternal and Child Health Programs, Washington, DC USA

**Keywords:** Mentorship, Workforce, Culture

## Abstract

**Introduction:**

Mentorship should be a transformative experience that propels mentees from one point in their career to another and drives personal growth. Within the field of maternal and child health (MCH), it is considered a critical professional duty. However, MCH has yet to explicitly embrace mentorship practice as a means to address workforce challenges including turnover, knowledge loss, and undue burden on the part of historically oppressed individuals and communities to overturn oppressive systems.

**Call to Action:**

We advocate for public calls for diversity and equity to be met with strategic enhancement of the practice of MCH mentorship. Transformative MCH mentorship should be used to promote positive identity formation, understanding of self in context, efficacy, and sustained commitment to working with MCH populations in ways that are inclusive and prevent the perpetration of the problematic power dynamics that lead to inequitable outcomes.

**Recommendations:**

We present recommendations to strengthen MCH mentorship practice. At the individual level, there should be a refreshment of norms and expectations, where mentorship is seen as a uniquely flexible opportunity for mutual learning. At the organizational level, embedding mentorship in all aspects of practice helps establish and sustain a culture of belonging. This transformative organizational culture can attract and retain future generations of professionals that are not only more representative of the populations that MCH programs support but are prepared to authentically elevate the needs and strengths of those populations. These suggestions incorporate best practices from other fields and include ideas for the MCH field in particular.

## Significance Statement

Mentorship is a critical practice for MCH professionals that facilitates knowledge sharing and builds the leadership capacity of both mentors and mentees. In spite of this, the MCH field is facing a workforce crisis and continues to grapple with how best to center equity in and across programs.

We advocate for the adoption of transformative approaches to MCH mentorship to address inequitable power dynamics in leadership that are linked to bias and that ultimately contribute to population-level disparities. Revitalizing MCH mentorship can contribute to the long-term advancement of anti-oppressive workplaces and, ultimately, anti-oppressive MCH policies to eliminate MCH disparities.

## Introduction

Mentorship is a strategy that effectively builds a pipeline for diversity in the maternal and child health (MCH) workforce (Kuo et al., 2015). It is critical to professional development and is separate from but complementary to academic training. Mentorship is inherently flexible—it can be a formal process with facilitated matching of mentors and mentees or relationships that develop organically.

There are numerous definitions of mentorship, many of which center on the relationship between mentor and mentee to develop skills and competencies of the mentee (Dopson et al., 2017; Hamelin & Paradis, 2018). Haggard et al. (2011) define mentorship as a delicate balance between, “coaching, guidance, feedback, encouragement, and emotional support” for the mentee (Haggard et al., 2011). Further, mentorship may occur in multiple settings including academia (Hamelin & Paradis, 2018) to professional practice (Dopson et al., 2017).

MCH encompasses the life course in unique ways, and therefore MCH mentorship is relevant for public health more broadly. Investment in and practice of mentorship should be considered a duty and privilege among MCH leaders as a means to pass on knowledge. However, the MCH field is facing a looming workforce crisis that is complicated by inadequate mentorship framework and practice. This is in spite of the fact that mentorship is widely recognized as an essential skill and an important resource within MCH (HRSA MCHB, 2020). There is a prediction that the combination of an aging workforce and a lack of MCH-specific public health programs will result in an underdeveloped MCH workforce as leaders retire (Streeter, 2015).

MCH mentorship should also be used to ensure that future generations of professionals are more representative of and prepared to authentically elevate the needs and strengths of diverse MCH populations. In acknowledgement of this, federally funded pipeline programs have prioritized recruitment and retention of racially and ethnically diverse faculty and trainees. The Association of Maternal and Child Health Programs’ (AMCHP) most recently published MCH workforce analysis based on the 2017 Public Health Workforce Interests and Needs Survey (PH WINS, 2017) highlights an opportunity to increase diversity in MCH leadership to advance representation, be it cultural, linguistic, or other forms of representation, and that executives in particular require help incorporating, “health equity and social justice principles,” into programs (AMCHP, 2017). While few scientific papers describe the failings of mentorship in the MCH workforce, informal and formal channels illustrate these issues. For example, data collected and presented through Georgetown University’s National Center for Cultural Competence and the University of California-San Francisco describe challenges faced by members from racial and ethnic groups that have historically been underrepresented in MCH related to finding supportive mentorship that is grounded in cultural sensitivity and respect (Toretsky et al., 2018; Georgetown University National Center for Cultural Competence, 2020.).

## Call to Action

We call for renewed investment in mentorship of the MCH workforce. Importantly, we believe public calls for diversity and all forms of equity should be met with strategic enhancement of the practice of MCH mentorship. The practice of mentorship should be a transformative experience that propels mentees from one point in their career to another and drives personal growth for both mentor and mentee. Examples of newer models of mentorship described in the social work literature promote mutual growth between mentor and mentee, building trusting relationships, and enhancing diversity (Warren, 2005; Fox & Kang, 2019; Lipscomb & Tejeda, 2021).

As a professional field, we must commit to investing in *transformational mentorship*. Using the language from the transformational leadership framework where leaders and followers support each other to advance to a higher level of effort and impact, we suggest that transformational mentorship should similarly be defined as an investment in the relationship between mentor and mentee for mutual benefit. This will support the creation of a workforce that intentionally embraces diversity of experiences so that we can better serve communities most threatened by structural inequities. These practices will improve our ability to inclusively work with populations facing MCH crises, to prevent the perpetration of the problematic power dynamics that lead to inequitable outcomes, and ultimately to identify new solutions rather than “band-aids” to problems that have been allowed to exist for far too long. By elevating the skills of both mentees and mentors, we have the opportunity to address the inequitable power dynamics linked to unconscious bias that ultimately contribute to population-level disparities, including MCH outcomes across domains (Bass & Riggio, 2006).

The same isms that result from othering people based on intersecting identities, including but not limited to age, race, ethnicity, gender, gender identity, ability, language, citizenship status, cultural values, political stance, attractiveness, and academic experience, among others, play out in mentorship relationships and dynamics. Now is the time that we start thinking of effective mentorship as a strategy to talk frankly about those isms in MCH (Wyatt et al., 2019).

The time is long overdue for addressing these isms. The COVID-19 pandemic has forced us to reckon with the many ways that isms impact MCH (Diaz, 2020). Simultaneously, recent events in the United States have spurred the development of policies to call out racism as a public health issue, including a recently adopted American Public Health Association late breaker policy (American Public Health Association, 2020).

Health care professionals, health department staff, and university faculty are increasingly participating in anti-bias training, and we believe that a similar investment in MCH workforce capacity should be made through training MCH professionals to engage in culturally humble, anti-oppressive, and respectful mentorship practices and embrace this practice as essential to the fulfillment of their roles. Transformational MCH mentorship has the power to increase diversity of the MCH workforce, increasing the potential for community-rooted public health solutions to be more globally embraced and scaled. This aligns with HRSA’s Maternal and Child Health Bureau’s goal of preparing and empowering MCH leaders from diverse communities to promote health equity and wellness and reduce disparities in health and health care (HRSA MCHB, 2020). Sociologists have described the need to develop proactive strategies to improve access to higher education mentorship for individuals from identity groups that have been historically left out of decision-making spaces (Wyatt et al., 2019). We advocate for these changes at both the organizational and individual levels.

By establishing these types of organizational norms, we have the power to promote a culture of belonging and trust in relationships, which addresses multiple workforce challenges in the forms of turnover, knowledge loss, and undue burden on the part of historically excluded individuals and communities to overturn oppressive systems (Bissell, 2019). These same useful practices can also be applied when working and engaging with communities that have been systemically silenced and/or not invited to conversations where decisions impacting their lives are being weighed and made.

## Recommendations

Considering the dearth of evidence and documentation surrounding MCH mentorship, we call for investment in research studies and program evaluation to identify strategies for developing and maintaining mentoring relationships that deliver unique benefit for all participants and bend the systems in which they operate toward justice.

At the organizational level, we urge all MCH leaders to embed transformational mentorship into the professional culture in their organizations. As part of this reimagined model of MCH mentorship, we advocate for continuous monitoring and evaluation of our practices to ensure that we are building systems that embed dignity within and across our workforce support structures. Finally, we suggest that organizations aiming to build their MCH mentorship capacity should consider the following questions to build a robust transformational mentorship framework:

- For whom are we creating support systems that are conducive to transformational mentorship?
- Who are we raising up as leaders and experts worthy of providing and/or receiving mentorship?
- What are the relationship conditions that need to be in place for mentors and mentees to grow and develop?
- How are we systematically identifying and providing opportunities for mentees and mentors to have critical conversations about identity, power, equity, beliefs, etc. and obtain the knowledge and skills needed to be in positive, transformational relationships?
- How are we building organizational structures and holding them accountable to support and incentivize the time, effort, and commitment needed to nurture transformational relationships for mentors and mentees?

Since mentorship may occur in diverse settings, from academia (Hamelin & Paradis, 2018) to professional practice (Dopson et al., 2017), the context in which MCH mentorship occurs matters for strategic planning and implementation mentorship initiatives. We suggest that flexibility be incorporated to ensure that the framework can be adapted to effectively and appropriately meet each settings’ unique conditions.

At the individual level, there should be a refreshment of norms and expectations for both mentors and mentees. Mentorship should no longer be seen as a one-way street and solely focused on transferal of professional knowledge; it must be a transformational tool that promotes positive identity formation, understanding of self in context, efficacy, and a sustained commitment to MCH populations. Following lessons learned from colleagues in psychology, there must be a recognition of the mentee’s expertise of their own experience (Wyatt et al., 2019). Frank and open recognition of power, equality, competence, likeability, beliefs, assumptions, identity, and difference are important to understanding how these issues might be impacting the mentors’ perception of mentees, and vice versa (HRSA MCHB, 2020). MCH leaders must actively create spaces where brave and transparent discussions about sensitive subjects may be had between mentors and mentees (McLaughlin, 2010). We should reimagine mentorship relationships to rebalance power and affirm the value of both professional and cultural knowledge. Both parties should also approach mentorship as a dynamic and living relationship and hold themselves accountable to a standard of bi-directional learning (Waljee et al., 2020). To facilitate this change, interactive training programs for the current and emerging workforce should focus on improving the skills needed to engage as mentors and mentees in ways that truly value others’ identities and perspectives (Osman & Gottlieb, 2018).

Revitalizing MCH mentorship dynamics at the individual level will increase interpersonal connectedness and trust, which will be important for establishing trust in the workplace. Through this individual-level relationship development, we contribute to the long-term advancement of anti-oppressive workplaces and, ultimately, anti-oppressive MCH policies to eliminate MCH disparities.

Finally, we acknowledge that transforming our approach to and practice of MCH mentorship is not a silver bullet and that mentorship alone cannot drive diversity, cultural competence, and sensitivity in the workforce. MCH disparities are deeply rooted in structural barriers that drive inequitable career and health outcomes. Mentorship can and should be embedded in more global pursuits to eliminate these barriers—a willing investment by all participants in self, others, and wider community. We also recognize that transformational mentorship will be most effective when MCH professionals feel empowered and choose to invest in their own growth to practice transformational mentorship.
